# Perceived COVID-19 Threat and Reactions to Noncompliant Health-Protective Behaviors: The Mediating Role of Desired Cultural Tightness and the Moderating Role of Age

**DOI:** 10.3390/ijerph19042364

**Published:** 2022-02-18

**Authors:** Conrad Baldner, Daniela Di Santo, Marta Viola, Antonio Pierro

**Affiliations:** Department of Developmental and Social Psychology, Sapienza University of Rome, 00185 Rome, Italy; daniela.disanto@uniroma1.it (D.D.S.); marta.viola@uniroma1.it (M.V.); antonio.pierro@uniroma1.it (A.P.)

**Keywords:** desired tightness, COVID-19, health-protective behaviors, norm violation, compliance

## Abstract

The COVID-19 pandemic is a health crisis that requires individuals to comply with many health-protective behaviors. Following the previous literature, cultural tightness has been found to be a key mechanism to increase coordination in order to mitigate collective threats (e.g., COVID-19). In this study, we test a moderated mediation model to examine whether the perceived COVID-19 threat could intensify the extent of desired tightness (i.e., a personal desire for cultural tightness), moderated by age. Subsequently, we test whether this could intensify individuals’ emotional reactions to non-compliance with COVID-19 health protective behaviors. The study relies on a cross-sectional design, with a sample of 624 participants residing in central Italy (i.e., Lazio). The data were collected from February to October 2021. Questionnaires contained self-reporting measures of the perceived COVID-19 threat, desired tightness, and personal emotional reactions to non-compliance with COVID-19 preventive measures (e.g., wearing a mask). The results confirm that the perceived COVID-19 threat is associated with an increase in the desire for cultural tightness—and that this relationship was moderated by age—and, consequently, with intolerance for noncompliance with preventive behaviors. Additionally, both direct and indirect effects of the perceived COVID-19 threat on negative emotional reactions to noncompliance were significant; this indirect effect was larger at high (+1 SD) age than at low (−1 SD) age. Overall, this research provides some insight into how people can respond to the current pandemic threat, and how this may have implications for violating rules and regulations to keep contagion under control.

## 1. Introduction

The COVID-19 pandemic represents a massive global health crisis that has taken place for two years at the time of writing. A new coronavirus (COVID-19) emerged in late 2019 and has sparked a global pandemic starting in early 2020 [1]. In order to contain the spread of the virus, many countries had to impose various measures and citizens were required, among others measures, to wear protective equipment (e.g., masks), keep social distancing and hygiene regulations (e.g., frequent hand washing), stay at home as much as possible, avoid crowded spaces, and comply with nationwide lockdowns [2]. The outcome of the preventive measures is tightly connected with the conduct kept by each individual; despite increased regulations, as of the time of writing, the pandemic has caused several million deaths worldwide [3]. 

Hence, much psychological research has been invested in understanding how people respond to the pandemic. Exceptional pandemic-related requirements have had a major impact on people’s lives, generating a significant amount of stress and uncertainty [4,5] and serving as a real-world test of pro-sociality [6], as individual actions, such as the implementation of adequate preventive behavior, are key to containing the spread of COVID-19 [6].

In this paper, we focus on examining the impact of the perceived COVID-19 threat on the tolerance of others’ inappropriate pandemic-related behaviors (e.g., refusing to wear a mask) through an explanatory mechanism: the desired “tightness”, i.e., the desire for stronger rules and greater sanctions for non-compliance [7,8,9,10]. Following Jackson and colleagues [7], the degree to which individuals want their country to enforce tightness can vary from person to person regardless of their country-level tightness. The desire for tightness [11,12] is rather an individual’s preference that a given context is culturally tighter, as individuals can endorse a culture other than the one in which they live [7]. In this vein, research [7,11,12] has shown that, as the salience of the threat increases, people’s desire for tightness increases. According to Pepitone [13], the violation of socially accepted norms generally causes a negative emotional reaction (i.e., anger), not only in those somehow affected by the violation, but also in the “observers” of such violations. In recent times, the literature has focused its attention on the (non) compliance/adherence to recommended health behaviors [14,15,16,17,18] and the way in which people react to the violation of such behaviors is a topic of contemporary interest [19]. We have hypothesized that, as the desire for tightness increases, the disapproval of non-compliance should increase. We aim to empirically test this hypothesis. Additionally, as the pandemic poses a number of threats to people of different ages, we are interested in examining the role of age in increasing the desired tightness due to the COVID-19 threat. Age-related differences have been found in the risk of developing severe health outcomes [20], such as death [21], stress related to the anxiety of developing COVID-19 [22], and in how people perceive the costs of being affected by the COVID-19 pandemic [23,24]; in particular, as people age, they perceive higher costs of being infected with the virus, suggesting that they are aware of the increased personal health risks associated with the infection [23]. This further suggests that the age of individuals may influence their response to the COVID-19 threat, such that the relationship between perceived threat and personal desire for tightness could be moderated by participants’ age. In sum, the conceptual model we empirically test predicts that the perceived COVID-19 threat should intensify the desired tightness and this relationship should be moderated by age; in turn, the desired tightness should intensify negative reactions to others’ non-compliance with health-protective behaviors. We delve into our theoretical background below.

## 2. Threat, Tightness, and Implications

There is little doubt that the current COVID-19 pandemic represents an “ecological threat”, or a factor from the surrounding environment that threatens societal existence [7]. On the other hand, when societies face collective ecological threats, they tend to tighten their social norms and punishments in order to be more likely to survive [7,8,9,10]. The cultural variation in the strength of social norms (i.e., how clear and pervasive the norms are) and the degree of sanctioning within societies (i.e., how much tolerance there is for deviation from norms) place worldwide societies on a continuum between “tight” and “loose” [8,25,26], whereby tight societies are strict, formal, and disciplined, have clearly defined norms and impose severe sanctions on individuals who deviate from the norms; on the contrary, loose societies have a lack of formality, regulation and discipline, have norms expressed through a wide variety of alternative channels, and have a high tolerance for deviant behavior [8,10,26].

Culturally tight, as opposed to loose, societies often have a history of vast ecological threats, such as natural hazards, invasions, population density and pathogen outbreaks, which have led them to develop great coordination and strict adherence to social norms in order to survive [8,9]. As mentioned, cultural tightening is a crucial mechanism for overcoming crises [27]. Consequently, tight nations were found to have fewer cases and deaths per million than loose nations during the COVID-19 pandemic [27].

Moreover, the personal characteristics of the members of a society often mirror those of the larger society in which they live [8], whereby individuals living in tight cultures, compared to loose ones show greater cautiousness and dutifulness, greater self-regulatory strength (i.e., greater impulse control), and higher levels of needs for structure and self-control [8]. However, Jackson and colleagues [7] also point out that individuals can endorse greater tightness, or looseness, relative to the norm of where they reside and live; accordingly, support (or desire) for cultural tightness (versus looseness) is not the same as living in a tight (vs. loose) society: a person can live in a loose environment and desire a tighter environment, or vice versa [7]. 

As mentioned, when societies face societal threats, tight rules and punishments for people who deviate from norms may help them to coordinate in order to survive [8,10]. Likewise, correlational and experimental findings [7,11,12] showed that threat also affects the desire that rules be stricter in the face of threat. This finding has been consistently shown by as a number of studies. Jackson and colleagues [7] found that a perceived induced threat was associated with an increase in personal support, or desire, for tightness in both U.S. states and countries across the globe. Mula and colleagues [11,12] found that the specific perceived COVID-19 threat was associated with an increased desired tightness in Italy. Nisa and colleagues [28] found, in a worldwide sample, that the more people perceived a personal health risk, the more they supported strict health measures (i.e., support for mandatory coronavirus vaccination and mandatory quarantine). Qin and colleagues [29] found that talking about the COVID-19 crisis (i.e., salient threat) among team members in workgroups was positively associated with team cultural tightness in a Chinese sample. These findings suggest that when people feel threatened, such as during the pandemic, they may want greater coordination, strict rules and penalties for deviant behavior to get out of it. However, our specific dependent variable—emotional reactions to noncompliance with safety precautions—has not yet been studied.

Additionally, since cultural tightness implies a low degree of tolerance towards norm-violating behavior [10,26], a personal desired tightness should lead to less tolerance towards such behavior. In a certain sense, wishing for a cultural tightening in the place where one resides also means wishing for a greater coordination for survival, which above all takes place in compliance with preventive measures. We look at the effects of better coordination at the national level, with tight nations having a greater capacity to limit cases and deaths during the COVID-19 pandemic [27]. On the contrary, inappropriate behaviors pose a serious problem to societies because the behavior of each individual is fundamental in order to contain the spread of COVID-19 [6]. This is true even of very costly behaviors (e.g., long-term social isolation and self-quarantine), which serve the collective cause [23,30]. We could therefore expect the desire for tightening to increase the disapproval towards those who do not engage in this costly but necessary collaboration.

## 3. The Present Research

In the present study, we focus on whether people’s desired tightness may mediate the relationship between the perceived COVID-19 threat and emotional reactions to noncompliance with COVID-19 health-protective behaviors; we expect that the perceived COVID-19 threat may increase people’s desire for restrictions and sanctions where they reside, consistent with previous findings [7,11,12]; given that age-related differences were found in the perceived cost of COVID-19 infection and actual health risk [23,31], we also expect that participants’ age will moderate this relationship. Although research on COVID-19 has been very common since the start of the pandemic, to our knowledge this is the first attempt to study how socio-psychological variables can predict reactions to pandemic-specific non-compliance. In turn, we suggest desired tightness will be associated with an increase in negative emotions to others’ non-compliance with health-protective behaviors. Or, put more schematically:

H1 The perceived COVID-19 threat should intensify desired tightness;
H2 The above relationship should be moderated by age;
H3 The desired tightness should intensify emotional negative reactions to the others’ non-compliance with health-protective behaviors.

**Figure 1:**
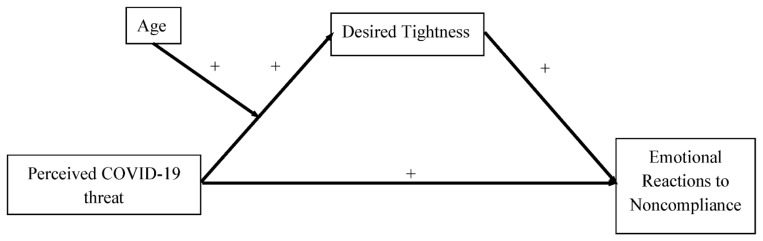
Hypothetical Model.

We therefore tested a moderated mediation model (see Figure 1) whereby the perceived COVID-19 threat (x) predicted negative reactions to others’ non-compliance with protective behaviors (y) through an increased desired tightness (m) moderated by age (w). The study is described below. This study has the potential to improve the literature in three ways (presented by order of importance): by adding to our knowledge of desired tightness during the COVID-19 pandemic in a location, Italy, not often studied by psychologists, but hard hit by the pandemic [32]; by studying if desired tightness is associated with emotional reactions to pandemic-specific safety precautions; and also by considering the role of participants’ age. Although this is a preliminary study, ascertaining if there is a direct and/or indirect effect of desired tightness with or without a moderating effect of age advances our knowledge of desired tightness and COVID-19-related reactions and provides a starting point for future research (e.g., interventions). In particular, we collected the data from the residents in the Lazio region, in central Italy, which has been very affected by infections, reporting, at the time of writing, a total of more than 400,000 COVID-19 cases [33,34]. 

## 4. Materials and Methods 

### 4.1. Ethics 

The study was approved by the Ethics Committee of the Department of Social and Developmental Psychology, Sapienza University of Rome (Prot. N. 15, 7 January 2021). 

### 4.2. Participants and Procedures

The data that we analyzed in this study were taken from a larger cross-sectional data collection, which was intended to measure tightness across Italy. The research was funded by Sapienza University; data were collected by the authors and collaborators. The current data were not previously analyzed. The eligibility criterion to participate in this research was to be at least 18 years old, so participants under the age of eighteen were excluded from the analyses. An additional eligibility criterion for the current research was for participants to reside in Lazio, a region of central Italy. Participants who indicated that they did not reside in Lazio were excluded from the analyses. The data were collected from February to October 2021. 

Participants were mainly recruited through online social networks (e.g., Facebook) and word of mouth initiated by the research collaborators. They were asked to complete a survey, which on average took 20 min, on an online link (sent via e-mail, WhatsApp or posted via social networks), which redirected the participants to Google Forms or Qualtrics XM. The survey was available through both platforms. Most of these participants (*N* = 620) received the survey’s link via Google Forms. Participation was voluntary. The informed consent specified that participants could exit the survey without penalty at any time. The survey was anonymous and participants’ IP address was not collected. We do not have information of how many participants left the research before completion. Respondents were prevented from responding to the survey multiple times via specific platform options.

A total of 624 participants residing in the central Italy (i.e., Lazio) volunteered in the study (69% females and 31% males). After giving their informed consent, participants completed an online questionnaire comprised of the set of measures described below, along with others that were not considered in this study, and presented in the following order (the same for all the participants): demographic information (gender (coded 0 = male; 1 = female), age, educational level, and occupation), perceived COVID-19 threat, desired tightness, and emotional reactions to non-compliance with COVID-19 health-protective behaviors. In order to limit the possibility of missing values, the responses to the items were mandatory, preserving the possibility for each participant to exit the survey at any time if not voluntary to respond. All study materials were presented in Italian.

### 4.3. Measures 

*Emotional Reactions to Non-compliance* were assessed with five items based on the most common COVID-19 health-protective behaviors in Italy (i.e., “Do not wear protective devices against COVID-19 (e.g., mask)”; “Little or no engagement in social distancing”; “Do not respect hygiene rules against the spread of COVID-19”; “Do not respect the lockdown when it is mandatory”; “Ignoring the restrictions against the spread of COVID-19”). Participants were asked to indicate their most likely emotional reaction (i.e., 1 = “approval”, 2 = “indifference”, 3 = “contrary”, 4 = “anger”, 5 = “violent rage”) in response to the above behaviors that could be carried out by others. The measure was inspired by Pepitone’s scale [13] and developed by the authors for this research. Items were averaged in a total score; internal reliability was high (Cronbach’s α = .91).

*Desired Tightness* was measured through an adaption of the Italian version previously used by Mula and colleagues [11]: the adaption consisted of desired tightness in one’s place (municipality) of residence, rather than country of origin. Specifically, participants were asked to answer five questions concerning the extent to which they think that their place (municipality) of residence should have the following characteristics at the time of answering, on a response scale anchored from “1” to “9” (e.g., “1 = Have flexible social norms,” “9 = Have rigid social norms”; “1 = Treat people who do not conform to norms kindly,” “9 = Treat people who do not conform to norms harshly”). Items were averaged in a total score of desired tightness; higher scores represented a higher desire for tightness for their place of residence; internal reliability was adequate (Cronbach’s α = .89).

*Perceived COVID-19 threat* was measured through four items from the Perceived Coronavirus Threat Questionnaire [35] (e.g., “Thinking about the coronavirus (COVID-19) makes me feel threatened”; “I am afraid of the coronavirus (COVID-19)”) (Cronbach’s α = .81). Answers were given on a 7-point Likert scale ranging from 1 (not at all) to 7 (totally). Additionally, participants were asked how concerned they were about the current coronavirus threat [11] on a 7-point Likert scale ranging from 1 (not at all) to 7 (totally). Items were averaged in a total score of concern with COVID-19; internal reliability was adequate (Cronbach’s α = .86). The original English items were translated and back translated by the authors, including native English and Italian speakers.

## 5. Results

Participants’ mean age was 32.24 (*SD* = 12.89, age range = 18–70 years). Table 1 provides the participants’ age distribution. As can be seen, most of our sample was young whereas only a small proportion could be characterized as “older adults.”

With regard to participants’ education, 5.8% had a middle school education, 45% had a high school education, 29.3% had a Bachelor’s degree, 18.9% had a Master’s degree, and 1% of participants had a PhD. With regard to participants’ occupation, 44.1% were students, 46.8% were workers, and 9.1% stated “other.”.

Descriptive statistics of the items and total scores of the variables of interest can be found on Table 2. Both kurtosis and skewness values are close to zero for each response item, appearing to not to exceed the recommended range of −1 to +1. [36]. Therefore, the pattern of responses would distribute normally [36].

Bivariate correlation and descriptive statistics can be found on Table 3. As can be seen, perceived COVID-19 threat was significantly and positively correlated with both desired tightness (*r* = .24, *p* < .001) and with negative emotional reactions to non-compliance (*r* = .35, *p* < .001), such that people who perceived COVID-19 more as a threat had more desired tightness and more negative emotional reactions. Desired tightness was significantly correlated with negative emotional reactions to non-compliance (*r* = .20, *p* < .001), such that people with more desired tightness had more negative emotional reactions. Further, age was also correlated with desired tightness (*r* = .12, *p* = .002), such that age and desired tightness increased together. 

**Figure 2:**
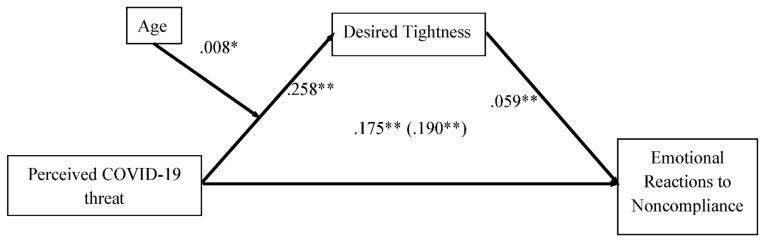
Moderated mediation results. Note. The total effect of perceived COVID-19 threat is in parentheses. All results are unstandardized. ** *p* < 0.01; * *p* < 0.05.

To further investigate these relationships, we conducted a moderated mediation analysis using the SPSS PROCESS macro (Model 7) [37] with 5000 bootstrap samples. Perceived COVID-19 threat, desired tightness, and emotional reactions to non-compliance were entered as the IV, mediator, and DV, respectively; in line with our hypotheses, age was entered as the moderator between perceived COVID-19 threat and desired tightness. Gender and education level were also entered as covariates. The results are displayed on Table 4 and Figure 2; all regression coefficients are unstandardized. As can be seen, the significant and positive relationship between perceived COVID-19 threat and desired tightness (H1; b = .258, se = .047, *p* < .001) was significantly moderated by age (H2; b = .008, se = .003, *p* = .022), such that this relationship was stronger at high (+1 SD) age (b = .36, se = .06, *p* < .001) than at low (−1 SD) age (b = .14, se = .06, *p* < .05). Further, there was a significant relationship between desired tightness and negative emotional reactions to non-compliance (H3; b = .059, se = .018, *p* < .001). Importantly, the indirect effect of perceived COVID-19 threat on negative emotional reactions to non-compliance was larger at high (+1 SD) age (b = .02, bootstrapped se = .008, 95% CI [.007, .042]) than at low (−1 SD) age (b = .008, bootstrapped se = .005, 95% CI [.001, .022]). 

We additionally performed a sensitivity analysis in order to see if the additional item in the perceived COVID-19 threat measure (i.e., How concerned are you about the current coronavirus threat?) had an inordinate effect on our results. We thus computed the perceived COVID-19 threat measure in a slightly different way, i.e., without this item. The results were equivalent to those from the original analyses. Additionally, in order to overpass the specificity of our sample’s characteristics (i.e., prevalently women and younger people), we tested the model in two sub-samples (i.e., only women and participants under 60 years old). In the female only subsample (*N* = 431), the significant and positive effect of perceived COVID-19 threat on desired tightness was significantly moderated by age, such that this relationship was only significant at high (+1 SD) age; the indirect effect of perceived COVID-19 threat on negative emotional reactions to non-compliance was significant at high (+1 SD) age and not significant at low (−1 SD) age. In the subsample composed by adults below 60 years old (*N* = 598), the significant and positive effect of perceived COVID-19 threat on desired tightness was not significantly moderated by age; the indirect effect of perceived COVID-19 threat on negative emotional reactions to non-compliance was significant at both high and low (−1 SD) age. These results are provided in the supplemental material.

## 6. Discussion

We examined whether perceived COVID-19 threat, moderated by age, could push desired tightness, and if this could in turn be related to emotional responses to others’ behavior about COVID-19 prevention. These results were supported; however, our participants were predominantly younger adults and our moderation by age effect was not present in the subsample that excluded older (60+ years) participants. This research is situated within the literature on the associations of tightness with variables specific to the COVID-19 pandemic [7,11,12,28,29]. Of these studies, only those by Mula and colleagues [11,12] recruited participants from Italy. Moreover, none examined emotional reactions to non-compliance to safety precautions during the pandemic—an important issue that socio-psychological research can help to illuminate. Although most people generally support public health precautions [38], health-protective behaviors (e.g., COVID-19 regulations) pose a “social dilemma” as they limit the freedom of the individual, but they pursue the cause of collective safety [23]. This suggests to us that, for effective pandemic management in terms of individuals’ responses (preventive behaviors), communities should negotiate social norms to find a balance between freedom and constraint, or “tight–loose ambidexterity” [10], depending on the level of threat. Nevertheless, this could serve as a starting point for future research that could examine how desired tightness could actually improve the prevention system to fight COVID-19. 

Importantly, the results we have obtained could undergo changes due to the evolution of the pandemic situation. Concern about the pandemic may have fluctuated in light of new preventive behaviors (e.g., vaccination) and this could affect both the desired level of tightness and intolerance towards others’ inadequate protective behaviors. Our data were collected about a year after the pandemic began, but the situation in Italy could continue to evolve. Hence, new investigations are needed considering the ongoing course of the pandemic.

We should recognize a number of the limitations of the present work that could be effectively addressed by future research to provide a more complete picture of the phenomenon: (i) our findings may be subject to common methods/source bias because they arise from cross-sectional surveys; (ii) the correlational nature of the data does not allow inferences to be made on the causality of the relationships found, which should be confirmed through longitudinal and/or experimental designs [7]; (iii) we may not have considered possible confounding variables, such as the participants’ experience of the threat (e.g., whether participants or someone they know contracted COVID-19 or how severely COVID-19 affected them or their place of residence) or a personal sensitivity to the threat (e.g., previously diagnosed diseases that could make infection more risky); (iv) the study was conducted on a non-representative sample of residents in central Italy, thus making it necessary to test the model in representative samples; (v) relatedly, it was a predominantly female and young sample; (vi) the results we obtained refer to the specific study period; and (vii) we focused on the individual level of variables (i.e., perceived threat and desired tightness). Although no significant differences were previously found [7] between tight and loose nations in personal support for tightness due to ecological threat, combining the study of group-level and individual-level predictors in multi-level designs would be particularly beneficial for further research. Finally, (viii) although we hypothesized and found that our results were stronger at higher age, COVID-19 can also be a large risk for younger people, and future research should also investigate if, in specific contexts, the concern for COVID-19 can predict interesting features among younger populations. 

## 7. Conclusions 

Despite some of the limitations that we recognized, the novelty of this research is related to its examination of the impact that this global crisis is having on the attitudes of individuals of different ages with respect to social norms and the respect for them. We hypothesized and found that the perceived COVID-19 threat interacts with age to predict the desire for tightness. Furthermore, we observed that desired tightness is associated with increased disapproval of others’ disrespect for health-protective behavior. Such behavior has previously been seen as protective for oneself and for the community as a whole [23] and is also a crucial way out of the pandemic situation. We believe it is important to empirically and further investigate the aspects that can potentially translate into adaptive responses of individuals [39,40] in the face of a serious threat, such as a pandemic. This research has implications both for future research and practice. Subsequent research could test the moderating effect of brief tightness manipulations on the relationship between perceived COVID-19 threat and our dependent variable. This could help to support our current findings and, if successful, could be used as a part of a communication strategy among populations heavily affected by the pandemic. For instance, these could be used in efficient and inexpensive social media campaigns. Moreover, we could also propose that individuals who are in more loose social environments could be targeted with information about the utility of safety precautions. Even though individuals can endorse a level of tightness that is not consistent with their place of residence [7], to make this more efficient we could target regions and municipalities that show evidence of looseness. 

## Figures and Tables

**Table 1:** Age distribution.

Age Range	Number of Participants out of the Total Sample	Percentage on the Total Sample
18–29	388	62.2%
30–44	108	17.3%
45–59	102	16.3%
60+.	26	4.2%

**Table 2:** Items and descriptive statistics of the Perceived COVID-19 threat, Desired Tightness and Emotional Reactions to Non-compliance with COVID-19 Health-protective Behaviors.

	*M*	*SD*	Min	Max	Skewness	Kurtosis
**Emotional Reactions to Non-compliance**	3.44	0.747	1.00	5.00	−0.525	0.762
Do not wear protective devices against COVID-19 (e.g., mask).	3.49	0.827	1	5	−0.549	0.674
Little or no engagement in social distancing.	3.41	0.796	1	5	−0.316	0.543
Do not respect hygiene rules against the spread of COVID-19.	3.29	0.827	1	5	−0.255	0.369
Do not respect the lockdown when is mandatory.	3.49	0.968	1	5	−0.485	-0.021
Ignoring the restrictions against the spread of COVID-19.	3.57	0.912	1	5	−0.582	0.489
**Perceived COVID-19 threat**	4.99	1.361	1.00	7.00	−0.429	−0.511
Thinking about the coronavirus (COVID-19) makes me feel threatened.	4.60	1.802	1	7	−0.389	−0.852
I am afraid of the coronavirus (COVID-19).	4.59	1.799	1	7	−0.346	−0.890
I am not worried about the coronavirus (COVID-19). (R)	4.78	1.875	1	7	−0.406	−0.981
I am worried that I or people I love will become sick from the coronavirus (COVID-19).	5.56	1.468	1	7	−0.863	−0.042
How concerned are you about the current coronavirus threat?	5.45	1.535	1	7	−0.964	0.423
**Desired Tightness**	6.28	1.585	1.00	9.00	−0.494	0.128
To what extent do you think that your place (municipality) of residence should have the following characteristics right now?						
1 = Have flexible social norms; 9 = Have rigid social norms.	6.07	2.038	1	9	−0.515	−0.232
1 = Treat people who do not conform to norms kindly; 9 = Treat people who do not conform to norms harshly.	6.29	2.045	1	9	−0.693	−0.050
1 = Have fewer rules; 9 = Have more rules.	6.21	1.801	1	9	−0.349	−0.144
1 = To be permissive; 9 = To be restrictive.	5.96	1.789	1	9	−0.239	−0.143
1 = Be tolerant of those who violate the norms; 9 = Be intransigent with those who violate the norms.	6.89	1.797	1	9	−0.895	0.508

**Table 3:** Bivariate Correlations.

	1	2	3	4
1. Age	-			
2. Perceived COVID-19 threat	0.01	(0.86)		
3. Desired Tightness	0.12 **	0.24 **	(0.89)	
4. Emotional Reactions to Non-compliance	0.08 *	0.35 **	0.20 **	(0.91)

Note. ** *p* < 0.01; * *p* < 0.05; Cronbach’s alpha on the diagonal.

**Table 4:** Moderated mediation analysis.

	Desired Tightness					Emotional Reactions to Non-Compliance				
	b	se	p	95%CI		b	se	p	95%CI	
				*LL*	*UL*				*LL*	*UL*
Age	0.014	0.004	0.002	0.005	0.023	-	-	-	-	-
Gender	0.301	0.138	0.029	0.029	0.573	0.025	0.063	0.691	−0.099	0.149
Education Level	−0.057	0.069	0.407	−0.194	0.079	0.007	0.031	0.813	−0.054	0.069
Perceived COVID-19 Perceived threat	0.258	0.047	<0.001	0.165	0.350	0.175	0.022	<0.001	0.132	0.218
Perceived Threat x Age	0.008	0.003	0.022	0.001	0.015	-	-	-	-	-
Desired Tightness	-	-	-	-	-	0.059	0.018	0.001	0.023	0.094
	*F* (5, 618) = 12.10, *p* < 0.001, *R^2^* = 0.089					*F* (4, 619) = 25.23, *p* < 0.001, *R^2^* = 0.1402				

## Data Availability

The data presented in this study are available on request from the corresponding author.
